# A Novel Application of Electroactive Polyimide Doped with Gold Nanoparticles: As a Chemiresistor Sensor for Hydrogen Sulfide Gas

**DOI:** 10.3390/polym11121918

**Published:** 2019-11-21

**Authors:** Lee Marvin G. Padua, Jui-Ming Yeh, Karen S. Santiago

**Affiliations:** 1 Department of Math and Physics, College of Science, University of Santo Tomas, Manila 1008, Philippines; marb8402@gmail.com; 2 Department of Chemistry, Research and Development Center for membrane Technology, Center for Nanotechnology, Chung Yuan Christian University, Zhongli, Taoyuan 32023, Taiwan; 3 Department of Chemistry, College of Science; Research Center for Natural and Applied Sciences, University of Santo Tomas, Manila 1008, Philippines

**Keywords:** conducting polymers, metal nanoparticles, gas sensor, hydrogen sulfide gas

## Abstract

This research paper presents a new application of electroactive polyimide doped with gold nanoparticles (PI/AuNPs) as a chemiresistor sensor for detecting hydrogen sulfide gas. The synthesis of PI/AuNPs was done in a simple 3-step process of polymerization using the as prepared amine-capped aniline trimer (ACAT), followed by imidization, and doping. Spectral analyses via FTIR, LC-MS and ^1^H-NMR confirmed the formation of amine-capped aniline trimer with a MW of 288 g mol^−1^. Comparison of ACAT, BSAA, and PI FTIR spectra showed successful polymerization of the last, while XRD validated the incorporation of metal nanoparticles onto the polymer matrix, showing characteristic diffraction peaks corresponding to gold. Furthermore, TEM, and FE-SEM revealed the presence of well-dispersed Au nanoparticles with an average diameter of about 60 nm. The electroactive PI/AuNPs-based sensor showed a sensitivity of 0.29% ppm^−1^ H_2_S at a linear concentration range of 50 to 300 ppm H_2_S (r = 0.9777). The theoretical limit of detection was found at 0.142 ppm or 142 ppb H_2_S gas. The sensor provided a stable response reading at an average response time of 43 ± 5 s, which was easily recovered after an average time of 99 ± 5 s. The sensor response was highly repeatable and reversible, with RSD values of 8.88%, and 8.60%, respectively. Compared with the performance of the conventional conducting polyaniline also doped with gold nanoparticles (PANI/AuNPs), the fabricated electroactive PI/AuNPs exhibited improved sensing performance making it a potential candidate in monitoring H_2_S in the environment and for work-related safety.

## 1. Introduction

Characterized by a pungent odor similar to the smell of rotten eggs, this colorless and flammable gas known as hydrogen sulfide (H_2_S) is considered poisonous at certain concentrations. As reported in Occupational and Safety and Health Administration [1,2], inhaling 20 ppm H_2_S may cause possible fatigue, loss of appetite, headache, irritability, poor memory, and dizziness. Depending on the length of exposure to H_2_S gas, severe conditions may occur when exposed to higher concentrations. For example, prolonged exposure to 150 ppm H_2_S gas could quickly paralyze the olfactory nerve disabling it from its capacity to recognize its presence. An hour of exposure to 700 ppm H_2_S gas could seriously damage the eyes, cause rapid loss of consciousness, or worst lead to possible sudden death [3,4,5,6].

Commonly, hydrogen sulfide gas is released in high quantities during excavation of landfills or swamps [7,8], and in higher amounts during a volcanic activity [9,10,11]. Furthermore, H_2_S gas is present in petrochemical reservoirs—in oil and natural gas wells, pipelines where unrefined petroleum is transported, and in refinery stations where H_2_S is removed [12,13,14]. In the paper industry [15,16], and iron smelters [17], H_2_S is a usual by-product. As H_2_S gas is denser than air, it can easily accumulate in areas like mine tunnels [18], sewers [19], and manure tanks [20,21]. Due to the ubiquity of H_2_S gas, and the possible danger this gas may cause, its detection is deemed highly important. However, the gas should not be directly perceived by the olfactory nose, nor should the level be closely assessed without the use of external devices.

There have been reports on the use of gas chromatography (GC) [22,23,24], infrared spectroscopy (IR) [25], ultraviolet spectroscopy (UV) [26,27,28,29], and fluorometry [30] in H_2_S determination, but these techniques involved the use of relatively expensive bulky instrumentation, and required tedious sampling preparations. Alternatively, the use of sensors was also a subject of heightened interest due to the many modes of detection that could be explored, such as electrochemical [30,31,32], optical [33,34], piezoelectric [35,36], and chemiresistive [37,38]. Sensors based on the chemiresistive mode was the simplest, operating based on the change in electrical resistance as the sensing material absorbs the gas analyte [39,40,41,42,43]. Metal oxide sensors (MOS) have been widely used in the construction of chemiresistive sensors of noxious gases due to their inexpensiveness and simplicity in both preparation and operation. These sensors also offer a wide array of detectable gases, hence expanding possible fields of applications. However, gas sensors fabricated using inorganic materials like metal oxides require operation at higher temperatures, as the high sensitivity of the said sensors is based on the high working temperature. This is often possible through the use of heated filament, and this entails the requirement of a higher amount of electricity to operate [44,45]. On the other hand, conducting polymers (CPs) also appear attractive as active elements in gas sensors. Compared with MOs, CPs present many advantages, such as high sensitivity, short response time, and room temperature operation. However, CPs offer low processability, and poor mechanical strength and chemical stability [46,47]. There have also been reports on the use of metal nanoparticles such as silver nanoparticles [26,48], and gold nanoclusters [30,49], but the detection is coupled to costly optical measurement devices.

This study, therefore, explored the use of combined electroactive polymers and gold nanoparticles to address issues encountered when these materials are used individually. Based on the literature survey conducted, this is the first time that electroactive polyimide decorated with gold nanoparticles has been used and developed for detecting trace levels of hydrogen sulfide gas at ambient room temperature. In the past, electroactive polyimide doped with gold nanoparticles was investigated for other applications such as electrochemical sensing of ascorbic acid [50]. Other studies of these combined materials focused on the effects of gold nanoparticles on polyimide in improving fluxes of the polymer for its possible nanofiltration application [51]. Most studies involving various uses dealt with EPI in different forms, while maximizing its inherent properties. Based on its redox properties, EPI was used as a recognition element in the fabrication of electrochemical sensors [52,53,54,55] and as an anticorrosion material [56,57,58,59,60,61,62,63,64]. Based on its doping and dedoping capability, EPI was also explored as a gas separation membrane [65], and as a smart material characterized for its switchable superhydrophobicity [66]. There have also been papers that investigated means to improve EPI’s mechanical strength [67,68] and thermal properties [69]. It is worthy of mentioning that the results obtained in this research showed that compared with the performance of the traditional conducting PANI/AuNPs, the fabricated electroactive PI/AuNPs exhibited improved sensing performance, making it a potential candidate in monitoring H_2_S in the environment and for work-related safety.

## 2. Experimental

### 2.1. Chemicals and Equipment

Aniline (99.0%, Fluka) was distilled under reduced pressure prior to use. Chemicals such as 4, 4’-diaminodiphenylamine sulfate hydrate (>97.0%, TCI), 4,4′-(4,4′-isopropylidene diphenoxy)-bis(phthalic anhydride) (BSAA, 97.0%, Aldrich), gold(III)chloride trihydrate (HAuCl_4_·3H_2_O, 99.99%, Alfa Aesar) 1-methyl-2-pyrrolidone (NMP, 99.7%, MACRON), *N*,*N*-dimethylacetamide (DMAc, 99.0%, MACRON, and 99%, Sigma-Aldrich, St. Louis, MO, USA), acetone (Ac, 99.5%, J.T. Baker, New Jersey, USA), methanol (MeOH, 99.8%, J.T. Baker), tetrahydrofuran (THF, 99.8%, RCI Labscan, Bangkok, Thailand), cyclohexanone (CH, 100%, J.T. Baker), dimethylformamide (DMF, 99.9%, J.T. Baker), acetonitrile (ACN, 100%, J.T. Baker) were used without further purification.

Infrared spectra were recorded using JASCO FT/IR-4100. To confirm presence of the Au nanoparticles, the samples were cut using a diamond knife to a 60–90 nm thick sections, and observed using a JEOL-200FX TEM (JEOL, Tokyo, Japan). The morphology of samples was recorded using scanning electron microscopy (SEM) (Hitachi S-2300). X-ray diffraction (XRD) analyses were recorded using a Philips X′pert Pro X-ray diffractometer. Electrochemical properties were investigated using a conventional three-electrode system connected to a VoltaLab 40 analytical voltammeter. ^1^H NMR investigations were carried out using Bruker 300 spectrometer, referenced to internal standard of tetramethylsilane (TMS). DMSO was used as solvent. LC-MS further facilitated analysis of samples using Bruker Daltonics ion trap mass spectrometer (Model: Esquire 2000 with an Agilent ESI source (Model: G1607-6001, Leipzig, Germany). 

A UNI-T UT71A multimeter with UT71A_B V3.00 software/Agilent 34410A desktop multimeter with Keysight BenchVue V 3.0 software (MatLab, CA, USA), was used in testing the gas sensing capability of the polymeric membranes.

### 2.2. Synthesis and Characterization of PI and PI/AuNPs

**Amino-capped aniline trimer (ACAT)** was prepared by following the procedure reported by Wei et al. Briefly, the synthesis of ACAT involved the use of ammonium persulfate to oxidize 2 equivalents of aniline and 1,4-phenylenediamine [70]. The success of ACAT preparation was confirmed by ^1^H NMR mass, LC-MS, and FT-IR spectroscopy.

The **electroactive polyimide (PI)** was synthesized using ACAT and BSAA. Solution A was prepared by dissolving BSAA (0.52 g, 1 mmol) in 8.0 g of DMAc with continuous stirring for 30 min at room temperature. Solution B was prepared by dissolving ACAT (0.29 g, 2 mmol) in 8.0 g of DMAc with continuous stirring for 30 min at room temperature. After stirring for 30 min, solution B was added to solution A, and mechanically stirred for 24 h to prepare a poly(amic acid) solution. The poly(amic acid)(PAA) was converted to polyimide(PI) by chemical imidization reaction. This reaction was done by adding acetic anhydride (0.102 g, 1 mmol) and pyridine (0.079 g, 1 mmol) to the solution, and refluxing for 3 h under nitrogen. Methanol (250 mL) was used to wash the PI and dried at 60 °C under vacuum for 1 day. The PI was characterized by ^1^H NMR, LC-MS, FT-IR spectroscopy, X-ray diffraction.

The **gold nanoparticle-doped electroactive polyimide (PI/AuNPs)** samples were prepared by dissolving PI in DMAc, and adding aqueous HAuCl_4_ solution (1 mM) at room temperature, then stirring for 6 h. After the reaction, centrifugal filtration was done to collect the precipitate. Large volumes of distilled water were used to wash the residue. The PI/AuNPs were dried in a vacuum oven at 60 °C. The PI-AuNP was characterized using TEM, FT-SEM, and X-ray diffraction.

### 2.3. Evaluation of Fabricated Sensors Toward H_2_S

**H_2_S headspace generation.** Hydrogen sulfide is generated in a reaction chamber by mixing 7.1 mg ferrous sulfide (FeS) and 10 mL 5 M hydrocholoric acid (HCl). This reaction produced 50 ppm concentration of H_2_S in a 55 mL sample chamber. The procedure was repeated to achieve 100, 150, 200, 250, and 300 ppm H_2_S.

**Figure 1:**
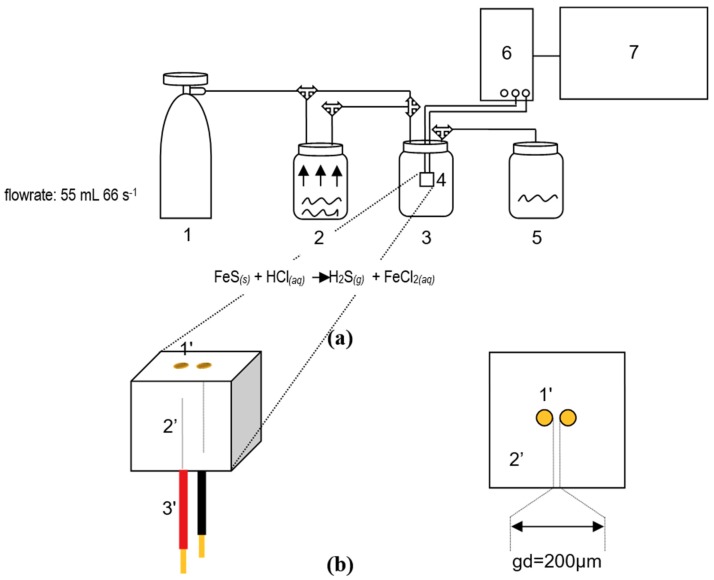
An illustration of the (**a**) gas sensing set-up: (1) N_2_ tank, (2) reaction chamber, (3) sample chamber, (4) PI/AuNPs-based H_2_S gas chemiresistor sensor, (5) reservoir for exhausted gas, (6) multimeter, and (7) computer; and (**b**) sensor substrate configuration: left-side view, and right-top view (1′) Au discs (end of Au wires), (2′) Teflon block, and (3′) insulated Cu wires.

As illustrated in Figure 1a, the gas sensing set-up was composed of a nitrogen tank connected to an enclosed reaction chamber (left, V = 55 mL), where generation of hydrogen sulfide gas occurred. The generated H_2_S gas was flushed into the sample chamber (middle, V = 55 mL) for determination using the fabricated sensor attached to a UNI-T multimeter. The resistance data obtained was recorded continuously using a computer. The gas exhausted from the sample chamber was bubbled in the third chamber containing water.

**Substrate preparation.** The substrate (Figure 1b) is composed of gold wires (18 karats, Ø = 0.5 mm, l = 0.5 cm) soldered to polished copper wires (Ø = 0.5 mm, l = 2.5 cm). A pair of soldered Au-Cu wires, separated by a 200 µm gap, was mounted into a custom made Teflon bar (10 mm × 10 mm × 4 mm). The wires were cleaned via ultrasonication in detergent solution for 15 min. This step was immediately followed with washing of wires using water, acetone, methanol, water, and then dried. The wires were held in a sturdy and upright position by applying a cyanoacrylate adhesive (Mighty Bond™ by Pioneer ® adhesives). The exposed Au ends, which served as the substrate, were subsequently polished with high grade of abrasive paper (1200 grit) and pad with aluminum slurry (Ø = 3 µm). The polished electrodes were then washed with methanol, sonicated in ultrapure water for 5 minutes, and dried.

**Sensor performance evaluation.** The sensor performance was evaluated based on sensitivity, linearity, linear concentration range, limit of detection, dynamic response and recovery characteristics, repeatability and reproducibility, and selectivity.

## 3. Results and Discussion

### 3.1. Synthesis and Characterization of PI-Based Materials

#### 3.1.1. Synthesis of electroactive polyimide-based materials

**Scheme 1:**
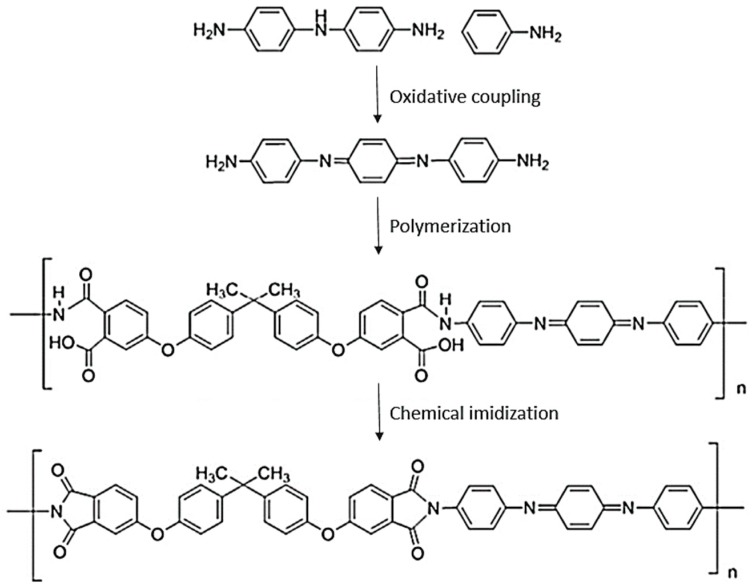
Proposed mechanism for the synthesis of electroactive polyimide.

The ACAT was synthesized by the oxidative coupling of 4-4′-diaminodiphenylamine and aniline monomer by the addition of ammonium persulfate as the oxidant (Scheme 1). The synthesized ACAT was then subjected to chemical polymerization with the aid of BSAA, resulting in the formation of poly(amic acid). Finally, the poly(amic acid) underwent chemical imidization by acetic anhydride and pyridine to form the final product, electroactive polyimide.

**Scheme 2:**
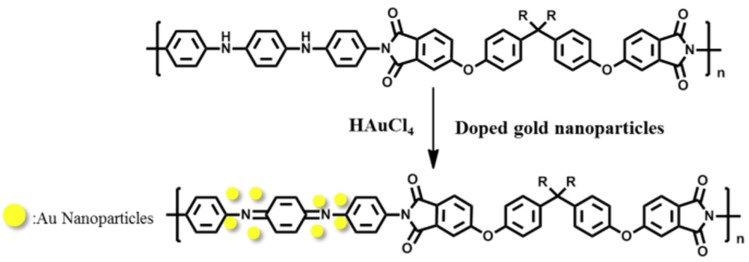
Proposed mechanism for the synthesis of gold nanoparticle-doped electroactive polyimide.

Scheme 2, adapted from the work of Ji et al. [50], shows the proposed mechanism of the formation of electroactive PI/AuNPs by doping gold nanoparticles onto the synthesized PI, with the aid of dimethyl acetamide and ethyl acetate solvents.

#### 3.1.2. Structural and Morphological Characterization

**Figure 2:**
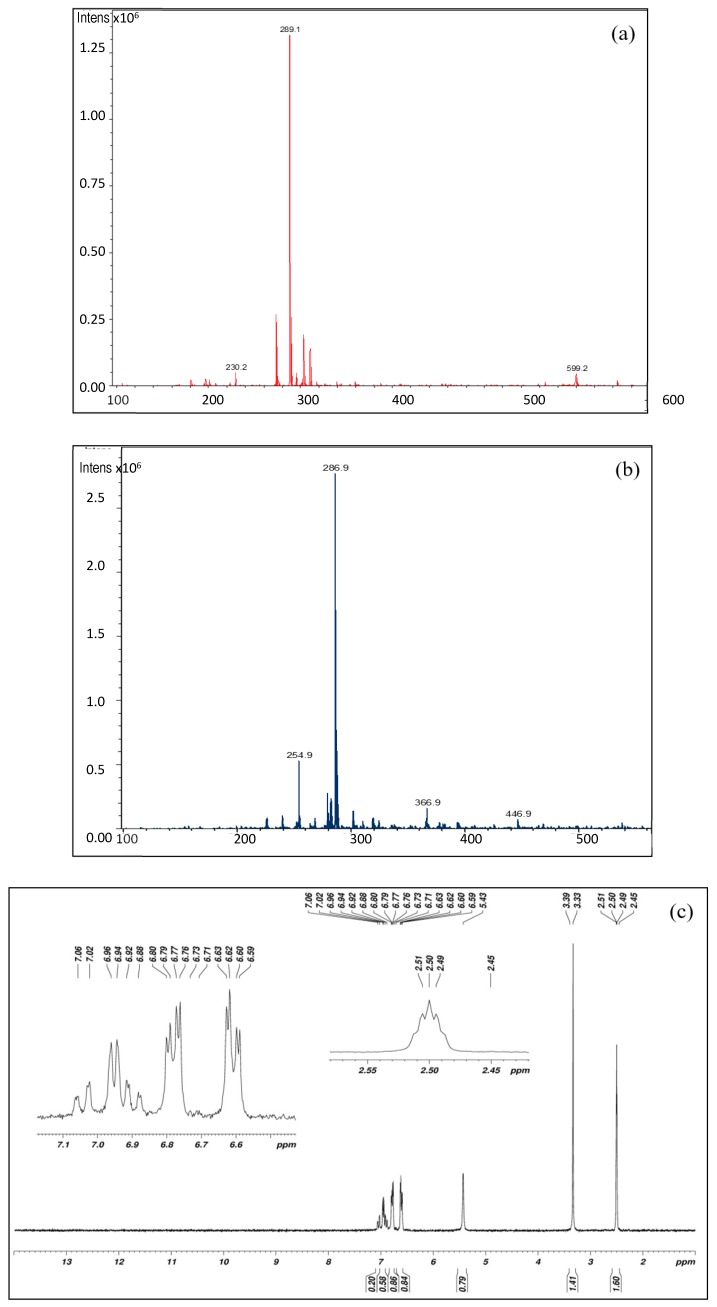
LC-MS spectra (**a**) MS^+^ mode and (**b**) MS^−^ mode, and (**c**) ^1^H-NMR spectrum of the synthesized ACAT powder.

Figure 2a,b shows the LC-MS spectra of the synthesized ACAT. It can be seen that the maximum intensity in the MS^+^ spectrum occurred at 289.1 m/z, while the maximum in the MS^-^ spectrum occurred at 286.9 m/z. These results suggest that the synthesized powder is ACAT. In theory, the molecular weight of ACAT is 288 g mol^−1^. The peak shifted by +/−1 which could be attributed to H. 

Further confirmation of the successful synthesis of ACAT using ^1^H-NMR spectroscopy was done. The sample used was prepared by dissolving a small amount of the synthesized ACAT in dimethylsulfoxide-d_6_((CD_3_)_2_SO). Figure 2c shows the spectrum obtained from the analysis.

**Figure 3:**
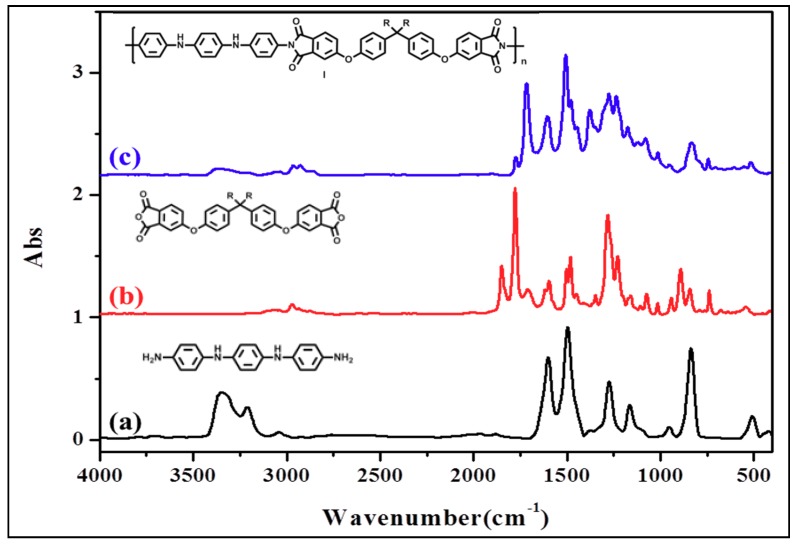
FTIR spectra of (**a**) synthesized ACAT, (**b**) BSAA, (**c**) synthesized electroactive PI.

The results show that PI (Figure 3c) has the same characteristic peaks with ACAT (Figure 3a) at 1504 and 1596 cm^−1^. The characteristic peaks at 1504 and 1596 cm^−1^ may be due to the vibration bands of benzenoid rings and quinoid rings of ACAT, respectively. At 1750 cm^−1^, PI (Figure 3c) and BSAA (Figure 3b) have characteristic peaks referring to the vibration bands of BSAA′s C=O groups. Based on the overall spectra, the synthesis of PI was successfully achieved.

**Figure 4:**
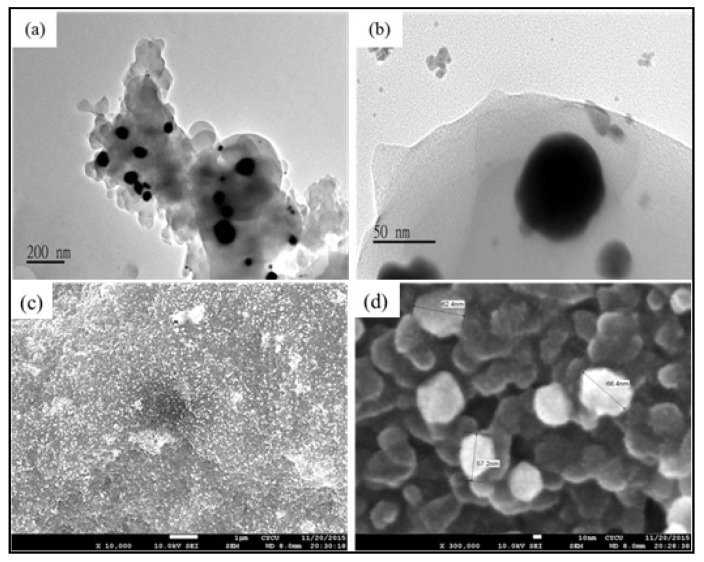
(**a**,**b**) TEM, and (**c**,**d**) FE-SEM images of the synthesized electroactive PI/AuNPs.

TEM and FE-SEM micrographs reveal the presence of well-dispersed Au nanoparticles with an average diameter of about 60 nm, as shown in Figure 4.

**Figure 5:**
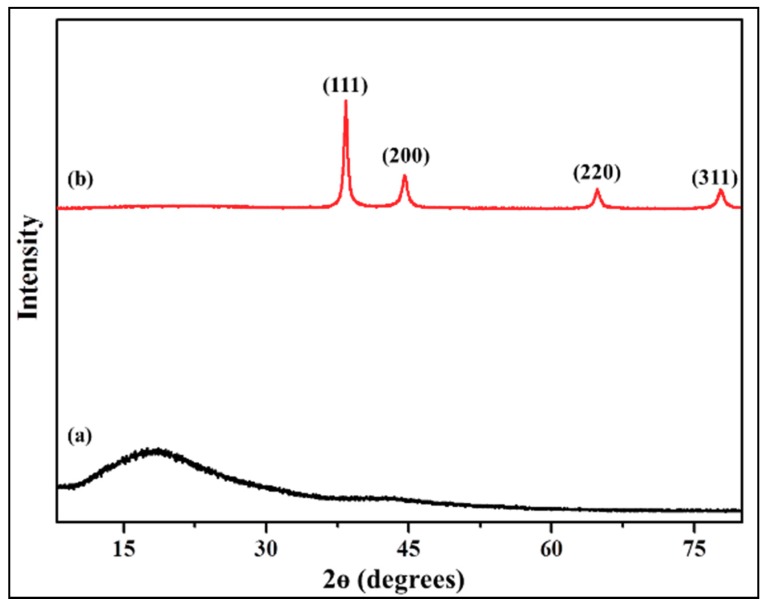
XRD pattern of electroactive (**a**) PI and (**b**) PI/AuNPs.

The diffractogram in Figure 5a shows a broad peak between 15–20° (2θ), which can be attributed to the semi-crystalline nature of the PI sample, having periodic parallel polymer chains. The diffractogram of the PI/AuNPs (Figure 5b) show the characteristic peaks of gold occurring at 38.01°, 43.96°, 64.50°, and 77.42° (2θ), corresponding to reflections from the planes, (111), (200), (220), and (311) [71]. The prominent diffraction at 38.01° revealed that zero valent gold grew and mostly fixed in the direction (111). This behavior also signifies the formation of pure gold nanocrystals [72].

### 3.2. Evaluation of the Sensor′s Performance

**Figure 6:**
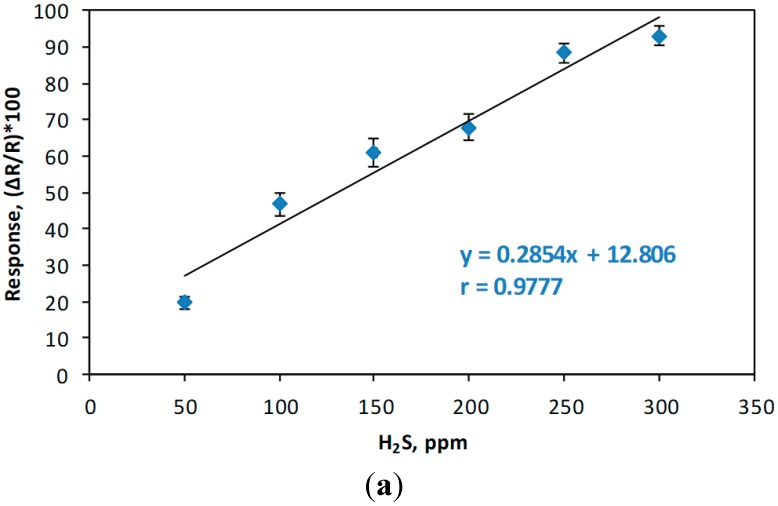

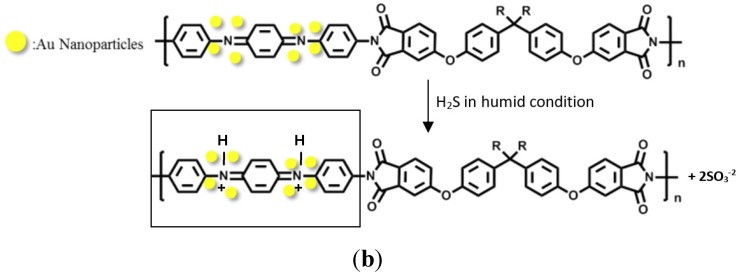
(**a**) Calibration plot obtained after the electroactive PI/AuNPs-based chemiresistor sensor was exposed towards increasing concentrations of H_2_S gas at RT (n = 3); and (**b**) the proposed sensing mechanism.

**Sensitivity, linearity, and limit of detection.** Shown in Figure 6a is the calibration curve constructed based in the performance of electroactive polyimide doped with gold nanoparticles (PI/AuNPs) towards increasing concentration of hydrogen sulfide gas. Responses were reported as (∆*R*/*R*_0_) × 100%; ∆*R*=/*R*_g_ − *R*_0_/, where *R*_g_ is the resistance of the chemiresistor gas sensor and *R*_0_ refers to baseline resistance. The sensor presented a sensitivity of 0.29% ppm^−1^ H_2_S gas and a linearity of 0.9777 at a dynamic linear concentration range of 50 to 300 ppm H_2_S gas. The theoretical detection limit was found to be 0.142 ppm or 142 ppb H_2_S, estimated according to the least-square method of fitting in the linear regime.

As indicated in Figure 6b, the decrease in resistance in the electroactive PI/AuNPs film could possibly be attributed to further doping of the polymer due to the sulfurous acid formed. Hydrogen sulfide gas (*K*_a_ = 1.1 × 10^−7^) in humid condition may generate sulfurous acid (*K*_a_ = 1.3 × 10^−2^), which consequently dissociates more effectively into hydrogen and sulfite ions. At room temperature and pressure, the hydrogen ions, then, protonate the polymer’s imine nitrogen sites of the ACAT chains. This phenomenon results to a corresponding increase in conductivity or decrease in resistance. Experimental data show that higher concentrations of H_2_S gas lead to higher decrease in resistance. It is speculated that the resulting positive charges in the imine nitrogen are counteracted by the negatively charged sulfite ions. Furthermore, the gold nanoparticles could possibly be contributing to the stabilization of the interaction by attracting the sulfur-containing counter ions toward the ACAT segments.

**Figure 7:**
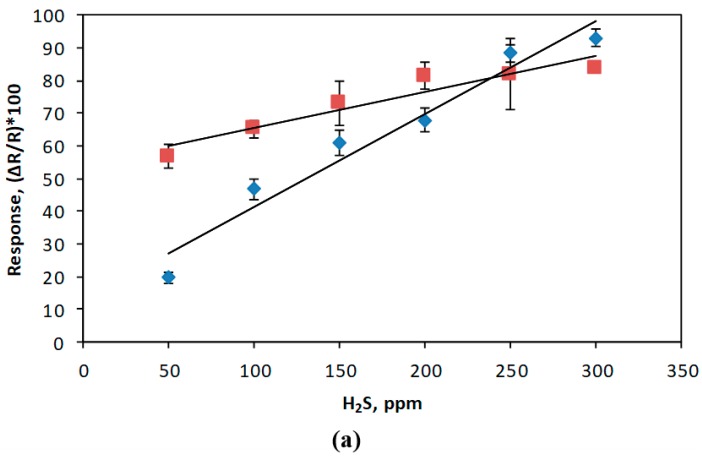

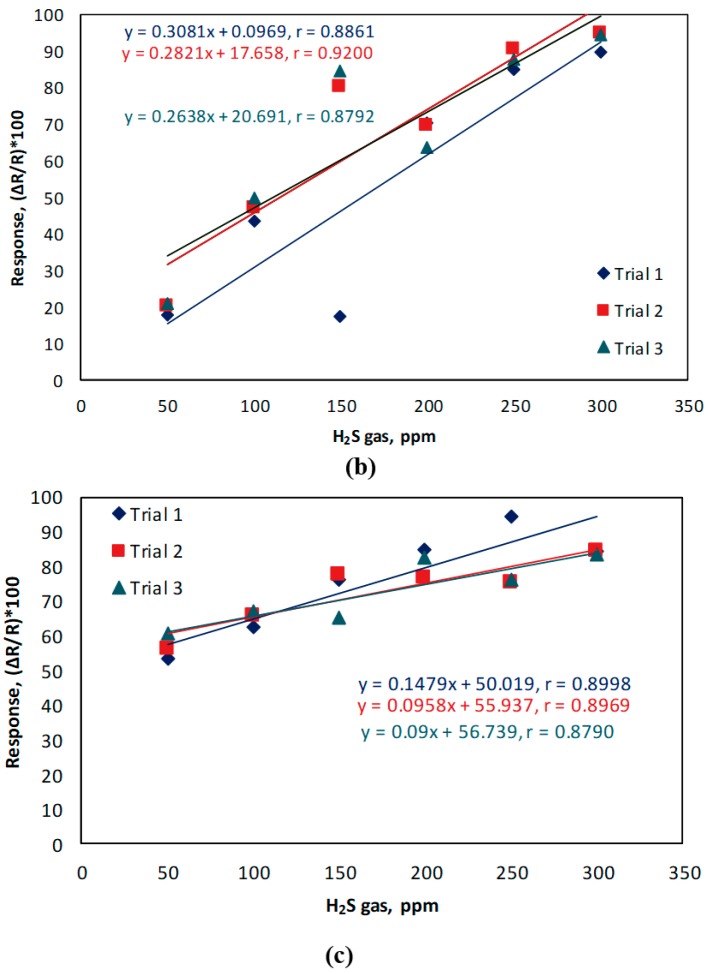
(**a**) A comparison of the calibration plots of electroactive PI/AuNPs-, and PANI/AuNPs-based chemiresistor gas sensors towards increasing concentrations of H_2_S gas (50 to 300 ppm, n = 3, RT). Repeatability studies based on the calibration plots of (**b**) PI/AuNPs, and (**c**) PANI/AuNPs.

**Repeatability of the sensor′s response.**Figure 7a shows a comparison of calibration plots of electroactive PI/AuNPs and PANI/AuNPs chemiresistor sensors towards increasing concentration of hydrogen sulfide gas. The latter has a sensitivity of 0.11% ppm^−1^ H_2_S, a theoretical detection limit of 0.45 ppm H_2_S, and a linearity of 0.9551 at the same linear concentration range of 50 to 300 ppm H_2_S gas. Based on sensitivity values, there was a 62% improvement in the performance with electroactive PI/AuNPs sensor. This may be attributed to the formation of additional conducting paths due to further doping of PI′s active sites. This, consequently, improved hopping of electrons throughout the polymeric chain.

The repeatability studies depicted in Figure 7b,c prove the better precision of all the trials, with respect to sensitivity values, in electroactive PI/AuNPs (% RSD = 8.88%) than PANI/AuNPs (% RSD = 28.36%).

**Figure 8:**
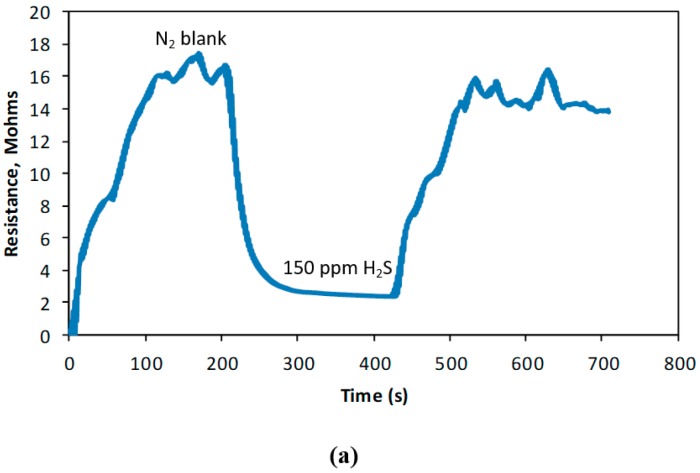

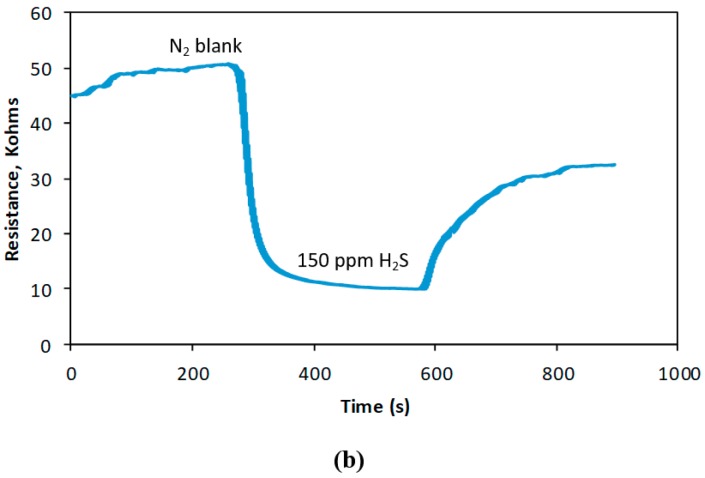
Dynamic response and recovery characteristics of (**a**) PI/AuNPs, and (**b**) PANI/AuNPs-based chemiresistor sensors towards 150 ppm H_2_S at RT (cycle = 1).

**Response and Recovery Characteristics of the Sensor.** Response time refers to the duration it takes the sensor to change from its initial state to its stable value, while recovery time is the period it takes the sensor to return to its initial state. As shown in Figure 8, a response time of 43 s and recovery time of 99 s was exhibited by PI/AuNPs while a response time of 55 s and recovery time of 103 s was shown by the PANI/AuNPs. The faster response time and recovery in PI/AuNPs could be attributed to the polymer′s three-dimensional (3D) surface, *i.e.*, the porous sensing membrane contributing to higher surface area (see FE-SEM presented in Figure 4d). This 3D structure, in both PI/AuNPs and PANI/AuNPs, allows easier diffusion of gas analyte into and out of the polymer matrix. Morphological studies revealed that PI/AuNPs are more porous than PANI/AuNPs. This increase in surface area could possibly be the cause of the higher response at a faster gas diffusion rate in PI/AuNPs. 

**Figure 9:**
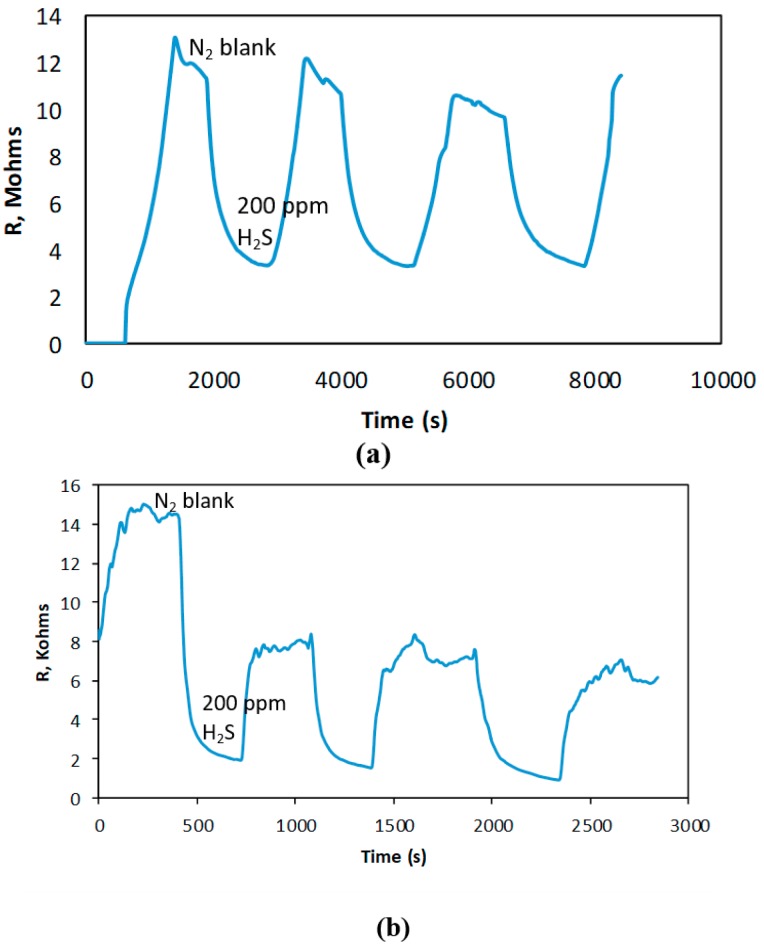
A comparison of the reversibility of the response of (**a**) PI/AuNPs, and (**b**) PANI/AuNPs-based chemiresistor sensors towards 200 ppm H_2_S at RT (cycles = 3).

**Reversibility of the Sensor′s response.** As shown in Figure 9a, the PI/AuNPs sensor exhibited a good reversibility within 3 cycles of exposure to alternating N_2_ blank and 200 ppm H_2_S (RSD = 8.60%). The reading returned to its baseline value once the gas analyte was removed.

The case is different compared with the performance of PANI/AuNPs sensor. Figure 9b shows that the sensor did not exhibit a good reversible process. The sensor experienced difficulty in reverting to the baseline reading after the first cycle of exposure to H_2_S gas. An RSD of 41.79% validated that PANI/AuNPs showed a poor reversibility.

**Figure 10:**
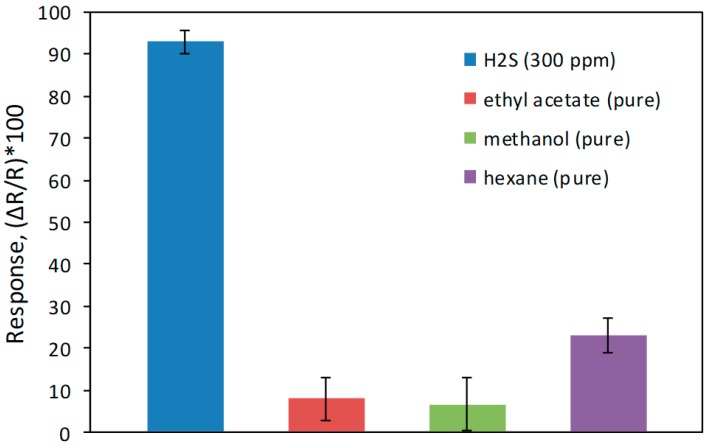
Selectivity test of electroactive PI/AuNPs with 300 ppm concentration of H_2_S, ethyl acetate (pure), methanol (pure), hexane (pure); (n = 3, RT).

**Selectivity.** The selectivity studies in Figure 10 revealed that the electroactive PI/AuNPs-based chemiresistor sensor resulted in the highest resistance change towards 300 ppm hydrogen sulfide gas compared to minimal to almost negligible response when the sensor was exposed to pure forms of solvents of varying polarity such as hexane, ethyl acetate, and methanol vapors. The same sensor, when exposed and tested towards the same concentration of ammonia gas, resulted in a signal that was opposite in direction, implying a different sensing mechanism. 

## 4. Conclusions

An electroactive polyimide/gold nanoparticles-based chemiresistor sensor that operates at ordinary room temperature was developed. The change in the electrical property, particularly the resistance of the sensing material, was used as a means to monitor the change in H_2_S gas concentration. Spectral studies confirmed successful synthesis of its sensing material. Morphological studies, on the other hand, confirmed the increase in surface area of electroactive polyimide after decorating it with gold nanoparticles. Compared with the conventional conducting PANI, likewise doped with AuNPs to ensure similar matrix, the electroactive PI/AuNPs exhibited an improved sensing performance. The proposed sensor offers an alternative means of monitoring hydrogen sulfide gas in a simple and inexpensive way.
